# Giant Colonic Diverticulum: Case report of a rare complication of a common disease

**DOI:** 10.1016/j.ijscr.2025.110909

**Published:** 2025-01-20

**Authors:** Usman Saeed, Therese Saunes, Ole Helmer Sjo, Johannes Schultz

**Affiliations:** a Department of Gastrointestinal and Pediatric Surgery, Oslo University Hospital, 0450 Oslo, Norway; b Department of Surgery, Volda Hospital, Norway; c Depertment of Gastrointestinal Surgery, Akershus University Hospital, Norway; d Institute of Clinical Medicine, University of Oslo, Norway

**Keywords:** Giant colonic diverticulum, Diverticular disease, Abdominal mass, Abdominal pain, Case report, Surgery

## Abstract

**Introduction and importance:**

Giant Colonic Diverticulum (GCD) is a rare but potentially life-threatening complication of diverticular disease, defined as a diverticulum larger than 4 cm, most commonly arising in the sigmoid colon. Its rarity could lead to diagnostic delays and mismanagement**.**

**Case presentation:**

A 64-year-old Caucasian female presented with persistent abdominal pain and abdominal swelling. Examination revealed a palpable mass in the left flank. A computed tomography (CT) scan demonstrated an 11x10x14 cm air-filled cavity adjacent to the sigmoid colon, initially misdiagnosed as contained perforated diverticulitis. Following a literature review, the diagnosis of GCD was considered. Definitive treatment involved a segmental sigmoid colectomy with en-bloc resection of the diverticulum. The postoperative course was uneventful, and the patient was discharged on postoperative day four with complete symptom resolution.

**Clinical discussion:**

The presentation of GCD can mimic common complications of diverticular disease, making diagnosis challenging. CT imaging is critical, typically showing a large air-filled cavity connected to the colon. Surgical resection is the treatment of choice to prevent serious complications such as perforation, abscess, volvulus, or malignancy. In this case, en-bloc resection proved effective, highlighting the importance of prompt surgical management.

**Conclusion:**

GCD is a rare and under-recognized clinical entity. Increased awareness is essential to ensure timely diagnosis and treatment. This case underscores the importance of integrating imaging findings, clinical judgment, and surgical intervention for optimal outcomes and emphasizes the need for further case documentation to aid in management strategies.

## Highlights

• GCD is a rare manifestation of diverticular disease, often diagnosed via CT imaging.
• GCD can be misdiagnosed as perforated hollow organ
• Presents with nonspecific symptoms, posing a diagnostic challenge for clinicians.
• GCD is an important differential diagnosis of abdominal masses
• Surgical resection is the preferred treatment to prevent serious complications.

## 1 Introduction

Giant Colonic Diverticulum (GCD) is a rare and distinct complication of colonic diverticular disease, characterized by a diverticulum that exceeds 4 cm in diameter. (1) GCD most commonly originates in the sigmoid colon, accounting for approximately 90 % of cases. (1) Though the exact incidence is unknown, approximately 200 cases have been reported, primarily in case studies or small case series. (2) Due to its infrequency and nonspecific symptoms, GCD is often misdiagnosed, leading to potential delays in appropriate treatment. The aim of this case report is to enhance clinical awareness of this rare entity and contribute to the limited body of literature on its presentation and management. The SCARE guidelines for reporting on surgical case reports were followed. (3)

## 2 Case presentation

A 64-year-old self-reliant Caucasian woman was referred to the community hospital following an outpatient CT scan that suggested a contained colonic perforation, initially interpreted as locally contained perforated diverticulitis. The scan had been ordered by her primary physician due to her complaint of persistent, low-grade left flank pain, which had progressively worsened over several months. The pain was described as constant, exacerbated by physical activity, and unrelated to eating or bowel movements. She also reported a gradual increase in abdominal distention over the past few months.

Her medical history was notable for breast cancer treated in 2004 with no recurrence, and well-controlled hypertension managed with medication. She lived independently and was fully functional in daily activities. At admission, she appeared well, with a height of 164 cm, weight of 64 kg, and a Body Mass Index (BMI) of 23.8 kg/m^2^. Vital signs were stable, with a tympanic temperature of 36.9 °C, and physical examination revealed a soft abdomen without peritonitis, but with a palpable, tender mass in the left flank. Rectal examination revealed no masses or visual/occult blood in the stool.

**Fig. 1:**
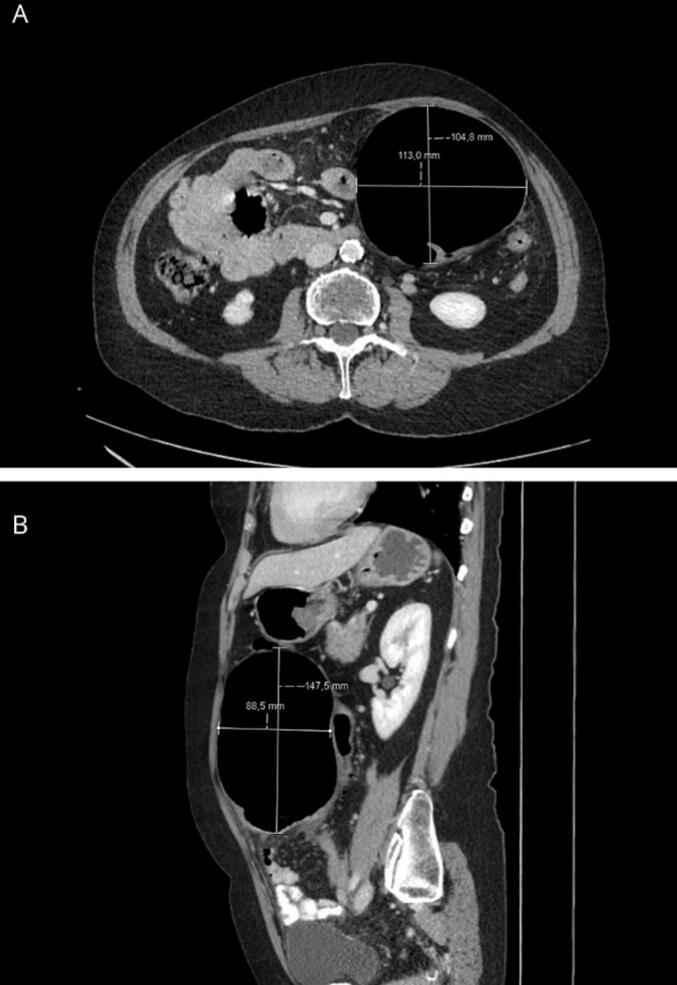
CT scan demonstrating a large, air-filled cyst adjacent to the sigmoid colon (Balloon sign). (A) Axial view, (B) Sagittal view.

Laboratory investigations revealed a hemoglobin level of 12 g/dL, a mildly elevated white blood cell count of 13.9 × 10^9^/L, and a C-reactive protein (CRP) level of 22 mg/L, suggestive of mild inflammation. A CT scan of the thorax and abdomen identified an 11x10x14 cm well-defined, air-filled cavity near the sigmoid colon, with surrounding fat stranding but no fluid (Fig. 1) The radiology report interpreted the air-filled cavity as free air outside the bowel lumen, suggestive of a contained perforation secondary to diverticulitis.

Given the patient's stable condition, oral antibiotic therapy with Amoxicillin (500 mg three times daily) and Metronidazole (400 mg three times daily) was initiated. A multidisciplinary team discussion between surgical and radiology teams maintained the initial diagnosis of contained perforation secondary to diverticulitis. Percutaneous drainage was considered but dismissed, as there was no evidence of fluid within the cavity. During this time, a PubMed literature search was conducted, raising suspicion of GCD as a possible diagnosis. This new information prompted reconsideration of the management plan, and the scheduled sigmoidoscopy was canceled due to concerns about the risk of perforating a dilated diverticulum.

**Fig. 2:**
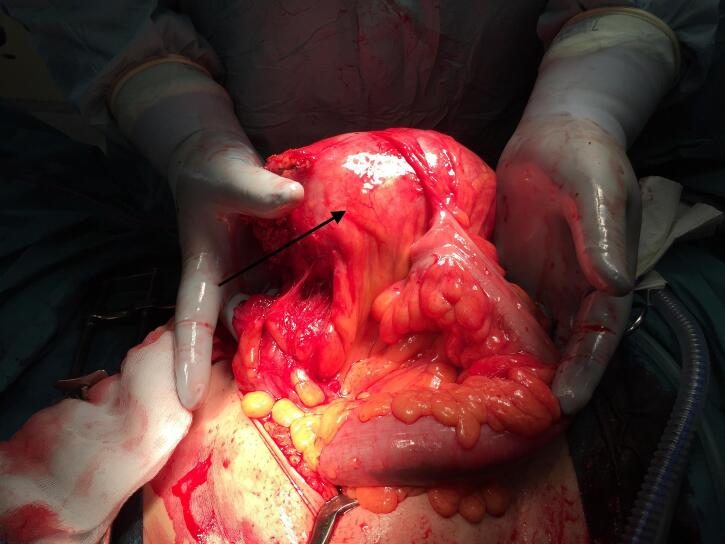
Intraoperative image depicting the giant colonic diverticulum (Arrow) adherent to the sigmoid colon.

**Fig. 3:**
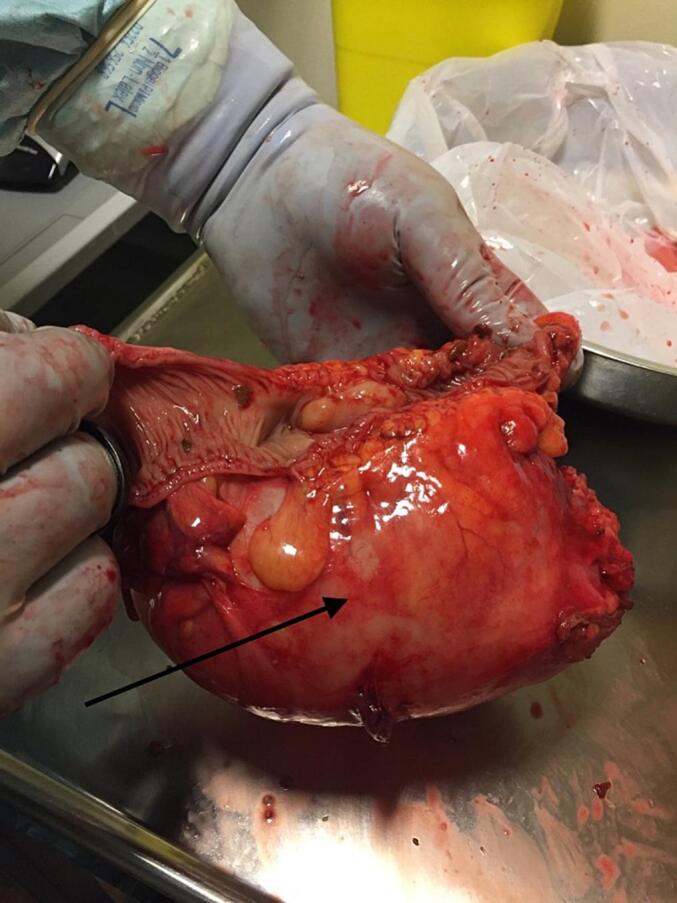
Resected sigmoid colon, opened along the mesenteric side, showing the large diverticulum (Arrow) attached to the colon on the anti-mesenteric side. Several smaller diverticula are also visible.

**Fig. 4:**
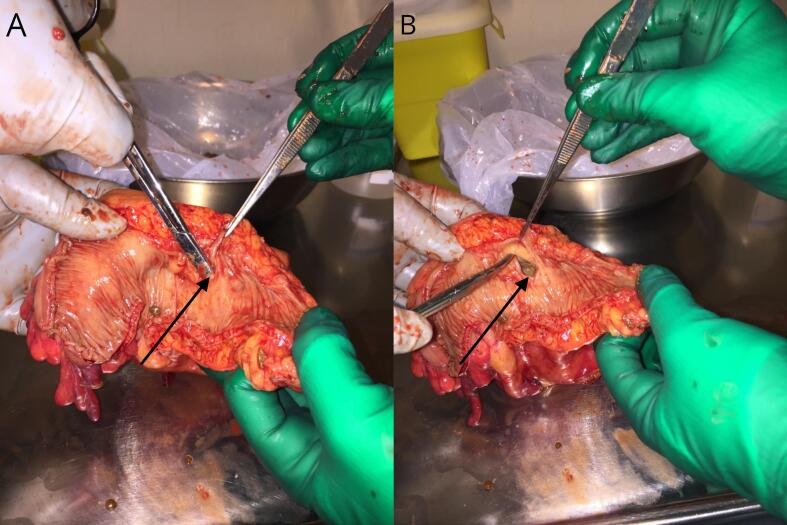
(A) A dissecting scissor inserted through a diverticular neck (Arrow) leading into the giant colonic diverticulum, demonstrating its narrow connection. (B) The diverticular neck is extended (Arrow), releasing pressure and deflating the giant colamnaunazaonic diverticulum.

As conservative treatment is not recommended for GCD, the patient was taken to the operating theater five days after admission for exploratory surgery, conducted by two consultants. A midline laparotomy was performed due to the large size of the lesion, and revealed a large diverticular mass originating from the sigmoid colon, adherent to the greater omentum, with no evidence of intraperitoneal contamination (Fig. 2). A Mini-Invasive approach was not considered feasible due the size of the lesion, which would require a large extraction site. After adhesiolysis, a segmental sigmoid resection with en-bloc excision of the GCD was performed, followed by a stapled, side-to-side anastomosis. The resected colon contained all visible diverticula, including multiple small diverticula (Fig. 3). Notably, one of the smaller diverticular openings communicated with the giant diverticulum. Upon inserting a dissection scissor into the narrow neck of this diverticulum, the trapped pressure was immediately released (Fig. 4). The postoperative period was uncomplicated, and the patient was discharged four days after surgery.

Histopathology confirmed an 9 × 6.5 × 3.5 cm inflammatory GCD with a pseudodiverticulum composed of fibrous scar tissue and lacking bowel mucosa, corresponding to a type 1 GCD as per McNutt's histological classification.

The patient was seen in clinic six weeks after discharge. She had no complaints and the symptoms prior to admission had resolved.

## 3 Discussion

This case highlights the diagnostic challenges posed by GCD, a rare and often underrecognized condition that can mimic other abdominal pathologies. Although colonic diverticulosis is common, GCD is an uncommon manifestation that often goes undiagnosed until complications arise or detailed imaging and a thorough literature review lead to a more precise identification.

GCD was first reported in the literature by Bonvin and Bonte in 1946, with the initial English-language description appearing in 1953 by Greene and Hughes. (1,4,5) Despite its association with diverticular disease, GCD is rare, affects men and women equally, and is usually identified in the seventh or eighth decade of life. (6) A systematic review by Nigri et al. in 2015 reported a mere 166 cases globally across 138 publications, emphasizing its rarity even within the context of a widely prevalent disease such as diverticulosis. (2)

The pathogenesis of GCD remains unclear, with two main theories proposed. The first is a “ball-valve” mechanism in which air becomes trapped within the diverticulum due to a narrow neck, resulting in progressive dilation of the diverticular cavity. (7) An alternative theory suggests that gas-forming bacteria within the diverticulum may contribute to its enlargement. (2) These mechanisms may operate synergistically in some cases, although further research is needed to confirm the specific processes involved.

Histologically, GCD can be classified into three types as proposed by McNutt et al. in 1988: (8).

1. *Pseudo-Giant Colonic Diverticulum*: Arises from gradual enlargement of a diverticulum without perforation and may contain remnants of muscularis mucosa or propria.
2. *Inflammatory Giant Colonic Diverticulum*: Develops secondary to a focal mucosal and submucosal perforation, resulting in the development of a fibrous scar cavity. This type, which accounted for our patient's case, represents the majority of GCD cases.
3. *True Giant Colonic Diverticulum*: A rare type that contains all the normal layers of the bowel wall, including serosa, muscularis, submucosa, and mucosa.

Another classification system by Choong et al. simplifies GCD into two types, with type 1 as a pseudodiverticulum related to diverticular disease and type 2 as a true diverticulum. (9) The histological findings in our case are consistent with type 1 GCD as per Choong et.al, an inflammatory pseudodiverticulum.

GCD presents variably and can be classified into four clinical categories (1,2,10):

1. *Acute Presentation*: Mimics acute diverticulitis, presenting with abdominal pain, fever, symptoms of bowel obstruction, and rectal bleeding.
2. *Chronic Presentation*: Characterized by nonspecific symptoms of abdominal discomfort and distention.
3. *Complications*: Arises from complications such as perforation, abscess, volvulus, or ileus, reported in up to 35 % of cases. Development of adenocarcinoma within the GCD is reported in 2 %.
4. *Asymptomatic*: Detected incidentally during clinical or radiological evaluations.

Abdominal pain is the most frequently reported symptom of GCD, followed by constipation and a palpable mass. (1,2) Additional symptoms include diarrhea, nausea, vomiting and rectal bleeding, while on physical examination an abdominal mass is palpable in approximately 50 % of cases. (2)

CT imaging is the preferred diagnostic modality for GCD due to its high sensitivity and ability to detect colonic diverticula and potential complications. (1,6) A typical feature of GCD is the “balloon sign”, characterized by a large gas-filled rounded structure adjacent to the colon, as observed in our case (Fig. 1). (11) Plain abdominal X-rays may reveal a large gas-filled structure adjacent to the colon, (12) while ultrasound is generally unhelpful and may yield misleading reassurance. (13) Colonoscopy is contraindicated, as it may cause perforation due to increased pressure and a valve like function within the GCD. (2)

Conservative management of GCD is generally not recommended, as delayed treatment may lead to serious complications, including adenocarcinoma within the diverticulum. (1) While nonsurgical therapies, such as antibiotic treatment or percutaneous drainage, have been proposed for patients unable to undergo surgery, surgical intervention remains the standard of care. (2,14,15) Antibiotics can be used to treat an acute inflammation before delayed elective colectomy. (2) The preferred treatment is en-bloc resection of the GCD and adjacent colon, with primary anastomosis as performed in our case. (1,2,16) In high-risk or emergency settings, a Hartmann's procedure may be performed, although the literature indicates that segmental resection with primary anastomosis has the highest success rate. (1,2) A simple diverticulectomy has been reported in the literature; however, it is associated with a high rate of recurrence and leaks, making it a less favorable option for management. (1,13,16) In most published reports, a midline laparotomy has been utilized for access; however, laparoscopic access has been reported as a feasible alternative for this procedure. (16,17)

## 4 Conclusion

GCD is a rare but potentially serious condition that requires timely recognition and definitive management to prevent complications. Our patient presented with typical features of chronic GCD, but her diagnosis was delayed due to the condition's rarity and overlap with other complications of diverticular disease. GCD should always be considered in the differential diagnosis of abdominal masses, particularly in patients with a history of diverticular disease. This case adds to the available literature on GCD by emphasizing the importance of clinical awareness, thorough literature review and multidisciplinary collaboration in diagnosing and managing rare conditions.

## Authors contributions

US drafted the manuscript. TS obtained the patients consent. OHS, TS and JS revised the manuscript. All authors read and approved the final manuscript.

## Ethical approval

No approval from by the ethical committee has been sought as this case study is written for educational purposes rather than research, and data are collected as part of routine care. National standards have been followed.

## Guarantor

Usman Saeed

## Consent for publication

Written informed consent was obtained from the patient for publication of this case report and accompanying images. A copy of the written consent is available for review by the Editor-in-Chief of this journal on request.

## Source of funding

No funding is received.

## Declaration of competing interest

The authors declare that they have no competing interest.
